# Case Report: Successful treatment of verruciform acanthotic vulvar intraepithelial neoplasia with 5-aminolevulinic acid photodynamic therapy

**DOI:** 10.3389/fmed.2026.1949400

**Published:** 2026-10-07

**Authors:** Yitao Qian, Wenjing Zha, Jie Shen, Ke Li, Szeman Cheung, Yiwu Yu, Jianhua Huang, Ting Lyu, Fei Miao, Hongwei Wang, Lei Shi

**Affiliations:** Department of Dermatology, Shanghai Key Laboratory of Clinical Geriatric Medicine, and Shanghai Institute of Geriatric Medicine, Huadong Hospital, Fudan University, Shanghai, China

**Keywords:** 5-aminolevulinic acid, HPV-independent, photodynamic therapy, verruciform acanthotic vulvar intraepithelial neoplasia, vulva, vulvar intraepithelial neoplasia

## Abstract

Verruciform acanthotic vulvar intraepithelial neoplasia (vaVIN) is a rare, human papillomavirus (HPV)-independent, p53 wild-type intraepithelial precursor of the vulva, and standardized therapies for this disease are currently lacking. We report the case of a 68-year-old woman who presented with a 2-year history of multiple warty papules and vulvar pruritus and was diagnosed with vaVIN on the basis of histopathology (verruciform acanthosis with compact orthokeratosis, hypogranulosis, and superficial keratinocyte pallor with minimal cytologic atypia) and immunohistochemistry (HPV negativity, a p53 wild-type pattern, and non-block p16 expression) analyses. She underwent 5-aminolevulinic acid photodynamic therapy (ALA-PDT) and achieved complete clinical remission, with no recurrence during the follow-up period. To our knowledge, reports on the use of photodynamic therapy for vaVIN are scarce. This case suggests that ALA-PDT may be a promising alternative treatment option for vaVIN.

## Introduction

Verruciform acanthotic vulvar intraepithelial neoplasia (vaVIN) is an uncommon, human papillomavirus (HPV)-independent verruciform intraepithelial disease of the vulva characterized by acanthotic and/or verruciform architecture, altered squamous differentiation, minimal cytologic atypia, wild-type p53 expression, and negative or patchy p16 staining (1). Clinically, vaVIN presents as solitary plaques or exophytic warty lesions. Despite typically subtle cytologic atypia, vaVIN is a precursor of vulvar squamous cell carcinoma (VSCC) with a reported 10-year progression risk of approximately 28%; moreover, morphologic or molecular overlap with verrucous carcinoma suggests a spectrum in a subset of patients (2).

Because of its rarity and deceptively subtle histology, vaVIN is often underrecognized, and high-quality therapeutic evidence remains limited. Current management is largely extrapolated and not standardized; complete excision is still commonly recommended. However, postoperative recurrence rates can reach up to 44%, necessitating long-term surveillance and highlighting the urgent need to explore alternative therapeutic strategies. Here, we report a case of successful treatment of vaVIN with 5-aminolevulinic acid–based photodynamic therapy (ALA-PDT), suggesting its therapeutic potential as an alternative for vulvar precursors and cancers.

## Case presentation

A 68-year-old postmenopausal woman presented with a 2-year history of vulvar pruritus. A physical examination revealed a whitish plaque adjacent to the labium majus, with multiple verrucous, papillomatous papules on its surface, accompanied by scaling and peripheral hyperpigmentation (Figure 1A). The initial differential diagnoses included condyloma acuminatum and vulvar lichen sclerosus. Further history taking revealed that the patient was otherwise healthy, with no relevant sexual exposure or a family history of the disease. Given the markedly different therapeutic implications of these diagnoses, a biopsy of the verrucous lesion was performed. Histopathological results revealed a marked verruciform acanthosis of the epidermis with exophytic papillomatosis, compact orthokeratosis with focal parakeratosis, attenuation of the granular layer, and cytoplasmic pallor of keratinocytes in the superficial spinous layer. No significant nuclear atypia was identified, and there was a minimal lymphocytic infiltrate in the superficial dermis (Figures 2A,B). The result of immunohistochemical staining for HPV was negative (Figures 2C,D). A further immunohistochemical analysis demonstrated a wild-type p53 staining pattern confined predominantly to the basal layer and non-block-type p16 positivity (Figures 2E–H). Collectively, these clinicopathological and immunohistochemical findings supported a diagnosis of vaVIN.

**Figure 1:**
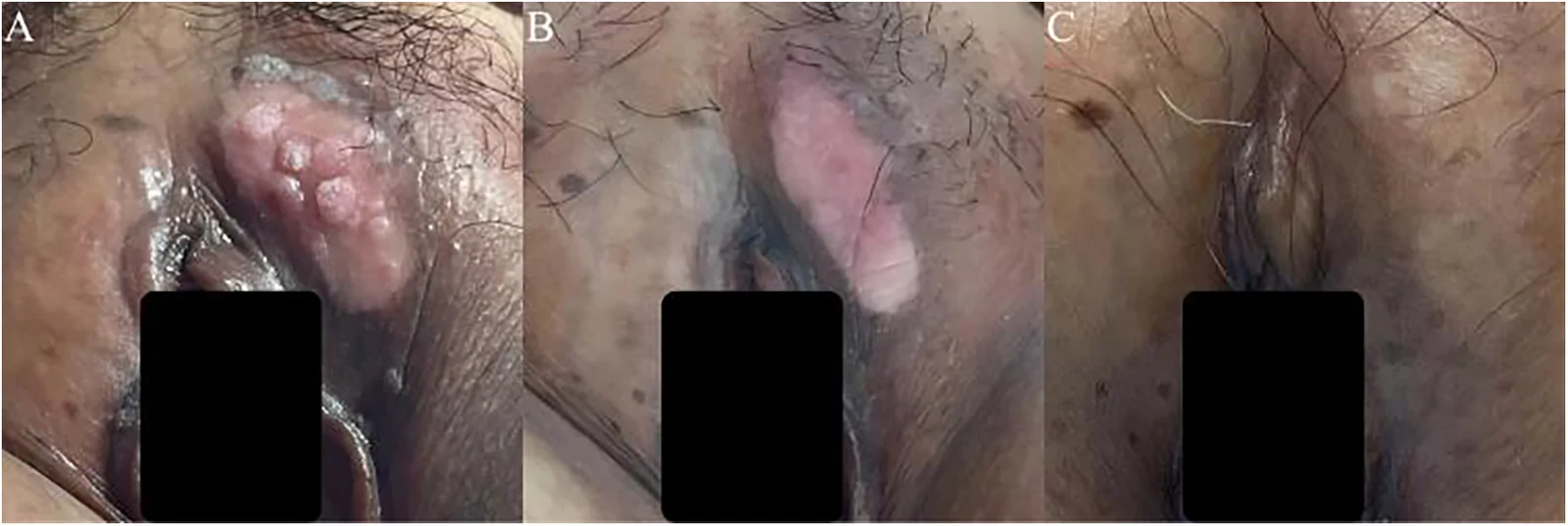
**(A)** Exophytic verruciform papules emerging within a localized white atrophic plaque adjacent to the labia majora. **(B)** After four treatment sessions, the papules had diminished, leaving a residual white patch. **(C)** Following all nine treatment sessions, both the white patch and papules had resolved, and the previously affected area became clear and smooth.

**Figure 2:**
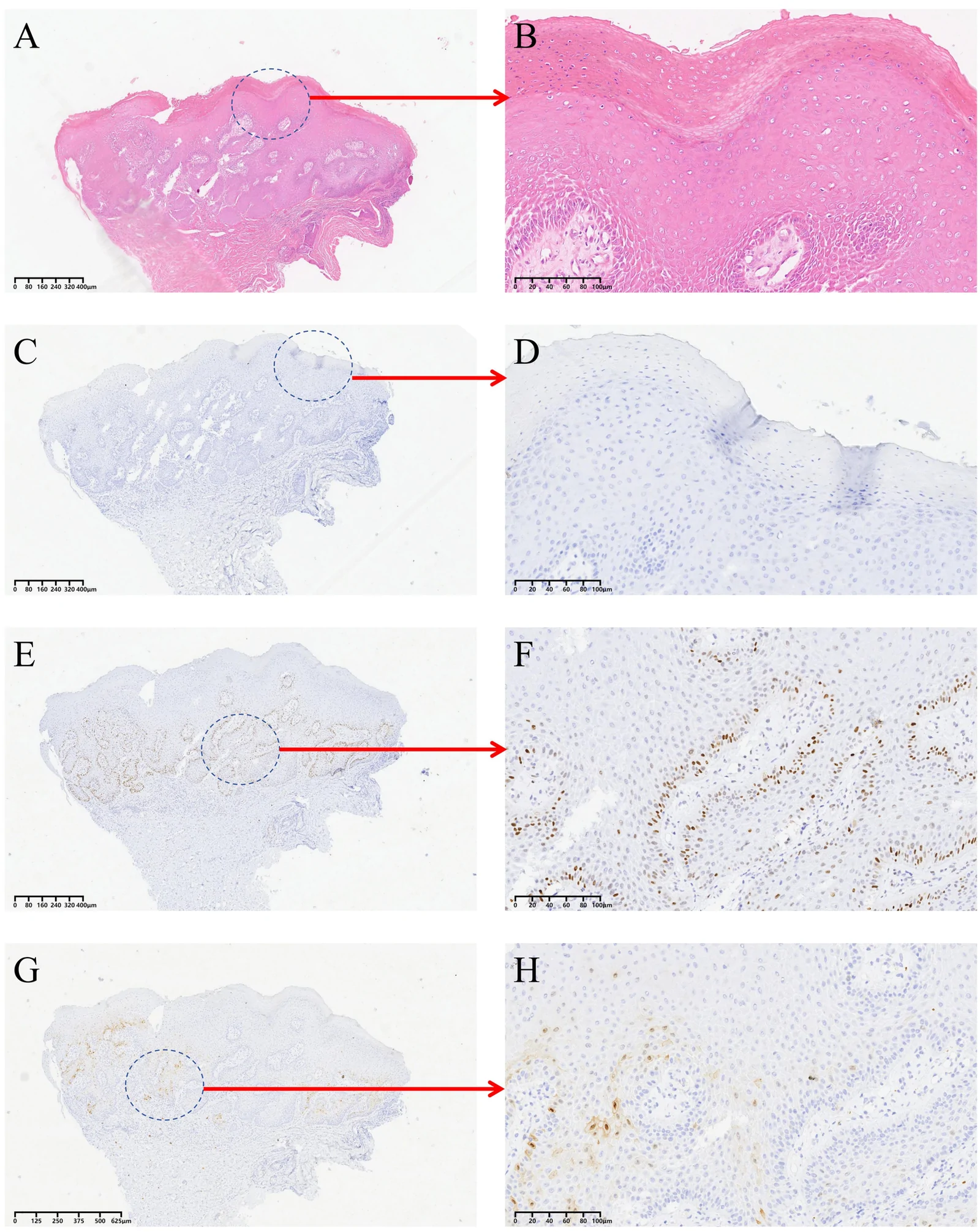
Histopathology and immunohistochemistry analyses. (**A**, 4×; **B**, 20×) Representative hematoxylin–eosin (H&E) staining images showing verruciform acanthosis with hypogranulosis and no significant atypia. (**C**, 4×; **D**, 20×) The result of a HPV immunohistochemistry test was negative. (**E**, 4×; **F**, 20×) p53 immunohistochemistry showed predominantly basal nuclear positivity (wild-type pattern). (**G**, 4×; **H**, 20×) p16 immunohistochemistry exhibited patchy, non-block positivity.

Given that the patient strongly declined surgery and preferred a tissue-sparing approach, ALA-PDT was selected as a conservative treatment. The treatment regimen was based on previously reported ALA-PDT protocols for vulvar squamous neoplasia, including VSCC and HPV-associated high-grade squamous intraepithelial lesions (HSILs).

The initial two sessions of ALA-PDT were performed once weekly, followed by seven sessions at 2-week intervals. During each session, ALA hydrochloride topical powder (118 mg/bottle, Fudan-Zhangjiang Bio-Pharmaceutical Co., Ltd., Shanghai, China) was used to prepare 20% ALA cream and was applied to the lesion under occlusion for 3 h, followed by irradiation with 635-nm red light at an irradiance of 80 mW/cm^2^ and a fluence of 144 J/cm^2^. Pain was assessed during illumination using a numerical rating scale (NRS; 0–10). A modified painless PDT was available as an alternative if the pain became intolerable. However, all NRS scores remained below 6, and the patient considered the pain tolerable; therefore, the patient did not require treatment interruption or conversion to the modified painless PDT protocol. Her vulvar pruritus reduced substantially during treatment, accompanied by progressive flattening and regression of the verrucous papules (Figure 1B). After nine treatment sessions, complete clinical resolution of both the papules and the underlying plaque was achieved, with restoration of a smooth, nearly normal-appearing skin surface (Figure 1C). No clinical recurrence was observed during 9 months of initial post-treatment follow-up, and in the most recent remote follow-up (3 years after treatment), assessment of patient-provided photographs showed no evident clinical recurrence. The patient remained symptom-free and reported marked satisfaction with the treatment outcome and improvement in her quality of life.

## Discussion

VaVIN shares morphologic features with several benign vulvar dermatoses and with other VSCC precursors, making careful clinicopathologic evaluation essential. Benign entities such as lichen simplex chronicus, psoriasis, and pseudoepitheliomatous hyperplasia typically present as diffuse, lichenoid flat plaques rather than as exophytic warty papules and lack the combination of superficial keratinocyte cytoplasmic pallor and hypogranulosis seen in vaVIN. Condyloma acuminatum is another common mimic. It is an exophytic verrucous lesion with koilocytosis and generally preserved basal maturation and is HPV-positive, thereby helping distinguish it from vaVIN. Among malignant precursors, the VSCC spectrum comprises HSIL and HPV-independent lesions, with the latter subdivided by TP53 status into differentiated vulvar intraepithelial neoplasia (dVIN; usually TP53-mutant) and vaVIN (TP53 wild type). These conditions can be distinguished by their morphology and immunophenotype: HSIL (especially Bowenoid papulosis and warty/basaloid variants) shows full-thickness atypia with block-type p16 positivity, and dVIN features basal or parabasal atypia with aberrant p53 staining (basal or diffuse overexpression, complete null pattern, or cytoplasmic staining). In contrast, vaVIN is HPV-negative, exhibits a p53 wild-type pattern (either scattered, heterogeneous nuclear staining in basal/parabasal layers or a mid-epithelial strong pattern with basal sparing), and shows p16 negativity or only patchy, non-block positivity (2, 3).

By analogy with related vulvar precancers and VSCC, ALA-PDT can be a rational therapeutic option for vaVIN (4, 5). Mechanistically, proliferating lesional keratinocytes preferentially accumulate protoporphyrin IX after ALA application, and subsequent red-light activation generates reactive oxygen species (ROS) that directly ablate these lesional cells. Beyond cytotoxicity, PDT orchestrates robust innate and adaptive immunity: neutrophils are rapidly recruited and activated (adhesion molecule upregulation and complement engagement); macrophages are stimulated by damage-associated molecular patterns (DAMPs) such as Hsp70, leading to proinflammatory cytokine release; and enhanced expression of MHC-I and NKG2D ligands augments natural killer (NK) cell activity and CD4^+^/CD8^+^ T-cell–mediated cytotoxicity (6). These molecular and immunological mechanisms may underlie the clinical efficacy of PDT.

Pain during illumination is the main adverse effect of ALA-PDT. To reduce treatment-related pain, we previously developed a modified painless PDT, which significantly reduced pain, while maintaining clinical efficacy in the treatment of condyloma acuminatum and acne (7, 8). However, given the premalignant nature of vaVIN and the potential uncertainty regarding efficacy associated with a shortened ALA incubation period, we adopted conventional ALA-PDT parameters in the present case as a cautious approach, with the modified painless protocol reserved for intolerable pain. Further studies are needed to determine the efficacy, safety, and optimal ALA-PDT parameters for vaVIN.

This study has several limitations. First, only a single patient was reported. To further establish ALA-PDT as an alternative treatment for vaVIN, its efficacy and safety must be evaluated in larger case series and well-designed clinical studies. Second, although no evident clinical recurrence was identified during the initial in-person follow-up or subsequent remote follow-up, histological clearance could not be confirmed because the patient declined a post-treatment biopsy. Vulvar precancerous lesions such as vaVIN carry a potential risk for recurrence and progression. Therefore, patients require long-term, regular clinical surveillance, with prompt biopsy of any persistent, recurrent, or suspicious lesion.

## Conclusion

In summary, this patient case suggests that ALA-PDT may be a feasible tissue-sparing option for carefully selected patients with vaVIN. Further studies are warranted to confirm its efficacy and elucidate the underlying mechanisms.

## Data Availability

The original contributions presented in the study are included in the article/Supplementary Material. Further inquiries can be directed to the corresponding author.
